# World Family Doctors Day 2019: Reflections from an African perspective

**DOI:** 10.4102/phcfm.v11i1.2139

**Published:** 2019-05-17

**Authors:** Robert Mash

**Affiliations:** 1 Division of Family Medicine and Primary Care, Stellenbosch University, Cape Town, South Africa

May 19th is World Family Doctors Day (WFDD), and this year the theme is ‘Family doctors – Caring for you for the whole of your life’.^1^ This editorial reflects on the relevance and meaning of this day and this year’s theme from an African perspective.

In sub-Saharan Africa, family doctors are scarce, mostly working in the private sector and without any postgraduate training in family medicine. Primary care in the public sector is mostly offered by nurses and mid-level doctors, known as clinical officers or clinical associates. Family physicians, doctors with postgraduate training in family medicine are even rarer, and in many countries non-existent or counted on the fingers of two hands.

In this context, the concept of medical generalism may be more appropriate, as it potentially encompasses all the different health professionals delivering primary care. ‘Medical generalism is an approach to the delivery of healthcare that routinely applies a broad and holistic perspective to the patient’s problems’…and involves ‘being able to deal with undifferentiated illness and the widest range of patients and conditions’ as well as taking ‘continuity of responsibility for people’s care across many disease episodes and over time’.^2^ Medical generalism, therefore, includes the principles of comprehensive and continuous care over the life course and embraces the theme of this year’s WFDD.

Although the principles of medical generalism should guide the training of all primary care providers, the reality is that many practitioners do not embody these principles.^3^ Training programmes may be too short or not sufficiently orientated towards principles of medical generalism. Often, primary care providers are trained in more algorithmic, selective and programmatic approaches to care that align more with public health priorities than family medicine. In the few countries that have studied the performance of primary care, the patients are not satisfied with the comprehensiveness^4^ and continuity of care.^4,5^

In the African context, family medicine can be defined as the subset of district health services provided by doctors (family physicians) with additional training in family medicine. In the public sector, family physicians are not the persons offering first-contact care and are often working as generalists in district or primary hospitals; their role, therefore, is different from family physicians in more highly resourced countries. Family physicians must not only be competent clinicians in all these settings but must also act as consultants to the health care team. They will need to develop the capacity of other health professionals and have expertise as clinical trainers. They will need to be skilled in clinical governance activities to improve the quality of clinical care and support health system reforms such as community-orientated primary care. Where family physicians are available, there is evidence that they are making a significant impact through all these roles and improving the quality of care.^6,7^

There are many stories of family doctors who have made a difference and gone the extra mile in caring for their patients. Wonca recognised Dr Atai Omoruto for her work in responding to the Ebola epidemic in West Africa.^8^ In South Africa, a recent media report told the story of Dr John Mitchell who hiked for kilometres with 25 kg of medicine and swam across a river to reach his clinic in the Eastern Cape when the road was blocked by protesters.^9^

Family medicine is slowly growing in sub-Saharan Africa, and we hope that the renewed international commitment to primary health care will include a commitment to family medicine.^10,11^ Primary care teams need the expertise that family physicians bring and district hospitals need people trained specifically for that setting who can fill the many skills gaps, particularly in rural and remote areas.

Countries in the region can be seen at different stages of change when it comes to family medicine. There are countries that have established family medicine training and have the potential to go to scale. Kenya, for example, now has five family medicine training programmes and South Africa has nine such programmes. There are countries that are just beginning to see family physicians emerge from training programmes, such as Botswana and Malawi, and there are countries that are starting to train family physicians, such as Zimbabwe and Zambia. Some countries are still contemplating family medicine, such as Tanzania, and there are attempts to advocate for its introduction.

There are, therefore, signs of hope and the potential for family doctors to contribute to and strengthen the primary care system, so that it can deliver on its promise to ‘care for you for the whole of your life’.
